# Rethinking Our Concepts and Assumptions About Autism

**DOI:** 10.3389/fpsyt.2022.903489

**Published:** 2022-06-03

**Authors:** Michael V. Lombardo, Veronica Mandelli

**Affiliations:** ^1^Laboratory for Autism and Neurodevelopmental Disorders, Center for Neuroscience and Cognitive Systems, Istituto Italiano di Tecnologia, Rovereto, Italy; ^2^Center for Mind/Brain Sciences, University of Trento, Rovereto, Italy

**Keywords:** autism, heterogeneity, precision medicine, diagnosis, subtype

## Abstract

Autism is a clinical consensus diagnosis made based on behavioral symptoms of early developmental difficulties in domains of social-communication (SC) and restricted repetitive behaviors (RRB). Many readily assume that alongside being optimal for separating individuals based on SC and RRB behavioral domains, that the label should also be highly useful for explaining differential biology, outcomes, and treatment (BOT) responses. However, we also now take for granted the fact that the autism population is vastly heterogeneous at multiple scales, from genome to phenome. In the face of such multi-scale heterogeneity, here we argue that the concept of autism along with the assumptions that surround it require some rethinking. While we should retain the diagnosis for all the good it can do in real-world circumstances, we also call for the allowance of multiple other possible definitions that are better tailored to be highly useful for other translational end goals, such as explaining differential BOT responses.

## Introduction

Nearly every article on autism tends to start off in the same way. “Autism is <*insert paraphrased DSM definition, or core symptom domains here*>”. Whether intended or not, this ubiquitous leading statement gives off the impression of an objective medical diagnosis. Because the diagnosis itself is automatically endowed with this face validity, it is uncommonly challenged by many. But perhaps we should heavily scrutinize and challenge it. Perhaps we need to persistently keep asking the tough questions regarding what validity it can claim to have and more importantly, whether one or many better definitions could exist. Over time, the landscape of autism has changed dramatically – from a once narrow to now wide definition, from being rare to now being common in the population, from something studied mainly in childhood to now being viewed across the lifespan, from something discrete to now something a bit more dimensional, from being one “autism” to many “autisms,” from pure to complex, and from “disorder” to “neurodiversity” (1). With all of this change over time, it should be perhaps expected that we do some rethinking on the concept and challenge ourselves in terms of our assumptions. Many in the field have already begun that discussion (1–18) and the dialogue should continue until we reach a revolution or paradigm shift that radically changes the situation to improve in areas that are currently heavily lacking, and which are most important given the objectives of the community and the field. In this perspective piece, we will contribute one drop into this ocean of “rethinking autism” from a zoomed-out perspective intended to primarily promote outside-the-box thinking on the topic. If we are to “rethink autism,” we should zoom out and not make many assumptions about what should be taken as fact and start asking very basic questions about the history of the label, what the diagnostic label was/was not intended for, and whether our current focus on some features rather than others may have led us astray. We conclude with an analogy about the concept of “trees” that may be useful in illustrating similar types of thinking and how we might rethink the topic.

## Back to the Future – the History of Autism

In moving forward, it may be useful to retrace your steps. A first way we can rethink autism is simply to look back at its history. For those who have just entered the field, this may be a difficult task, but there are several key references here which we think are essential reading on the topic (1, 19–27). We will not go through all the details here. However, to summarize one lesson that history has taught us so far, it is that “autism” is not a static concept over time, nor is it likely to be some objective “thing” out in nature waiting to be discovered and better understood. Rather, “what is autism” has changed considerably over time and will likely continue to evolve as we move forward. This notion of change in the diagnostic concept over time is important to be aware of, because we should not sit idly by assuming the current concept is necessarily more correct than past conceptions. Notions such as “prototypical autism” captured by Mottron and colleagues alongside the idea of weaning effect sizes over the years may support the idea that a previous conception of autism was more impactful (3, 4, 28). However, the sheer fact that the concept itself is non-stationarity should teach us to be highly skeptical of any current or past conception and any face validity that the diagnosis may be implicitly endowed with upon first glance. One way we might be able to evaluate whether the current situation is meeting our needs should be to question what the diagnosis is good for, but also what the diagnosis is not so good for. Certainly, we would not want to chuck out the diagnosis for all the good it does in real-world circumstances, but we should also not dogmatically hold onto it and resist change when we can all agree with its many shortcomings. Acknowledging the non-stationarity of the diagnostic concept itself, via a look back at history, should be the first step in being able to let go and allow for the possibility of new ways to characterize autism that fits the current zeitgeist and needs of the community and field.

**FIGURE 1:**
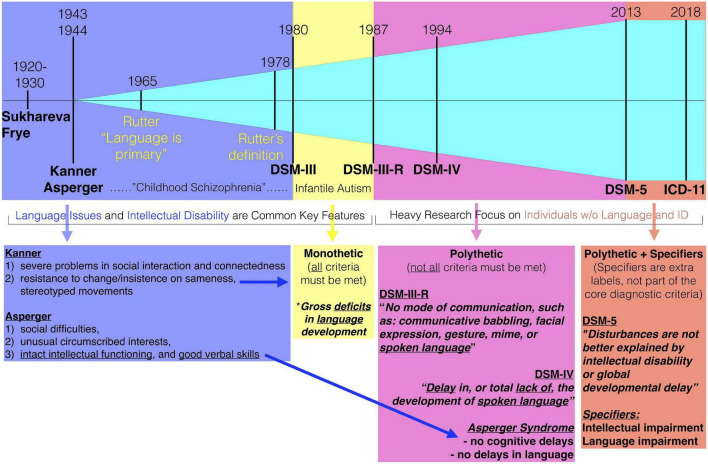
Highlighting the 1987 breakpoint of the change to DSM-III-R criteria. Before DSM-III-R, intellectual disability and early language issues were common key features. Even Rutter’s early opinions were that language was primary or core to autism. The change from monothetic to polythetic criteria in DSM-III-R changed all of this, since it allowed for individuals to be diagnosed without those kinds of issues. Over time, features such as intellectual disability and early language issues were filtered out altogether in the DSM-5 diagnostic criteria and are now used as specifiers. Star indicates one of the monothetic criteria regarding language.

Amongst the many notable key changes throughout the history of autism that could be commented on, here we isolate one specific change point that we believe is highly relevant for underscoring a major change in the population landscape of autism. In 1987, the DSM criteria changed from a monothetic (all criteria must be met) DSM-III to a polythetic (not all criteria need be met) DSM-III-R criteria. One of the most dramatic effects of this monothetic-to-polythetic shift was the sidelining of early language issues as a core and necessary feature. Before this point in time, influential individuals key to the construction of DSM-III criteria, such as Michael Rutter, had suggested that early language issues were a key feature of autism (29, 30). However, with the emergence of Asperger’s original case studies to the English speaking world (31), notable individuals such as Lorna Wing were influential in arguing that the concept of autism be broader than that of Kanner’s and DSM-III, particularly with respect to whether early language issues were essential. Wing also introduced the concept of the symptom triad (e.g., social, communication, and RRB) and the notion of a “spectrum” to further expand how social-communication difficulties might manifest in different types of individuals (e.g., aloof, passive, and active-but-odd) (32). Wing’s influence for this broader view (21, 33) were important factors in the DSM-III-R changes and to a polythetic relaxation which also made early language issues non-essential. All of these changes are likely important for giving us a broader and more complete view of the heterogeneous way that social-communicative issues can arise in different individuals. However, the impact of the change regarding the non-essential nature of early language issues cannot be understated. This change substantially reshaped how the autism population could be conceptualized – from once being a large majority of individuals with substantial intellectual and early language issues, to nowadays reflecting a large majority of autistic individuals without such issues (Figure 1). The impact of mixing together such individuals is still a prominent and current clinical issue. For example, as recognized by a recent Lancet commission, the label currently in use (mixing together all types of individuals) does not signify the differential need for services and support that the most profoundly affected individuals require (34).

We also note that at this point in time (i.e., mid 1980’s to early 1990’s), modern technologies that would allow us to peek into the underlying biology (e.g., high-throughput imaging and genome sequencing) were not available. Thus, these changes were made without the opportunity for science to sufficiently put to the test whether such a change was mixing together very different underlying neurobiology. Perhaps this last point could be argued to be irrelevant if someone were to claim that the diagnostic criteria was never made in the first place to differentiate individuals by biology. This is certainly true. However, given that the diagnosis is typically implicitly endowed with a certain type of face validity that most medical diagnoses also possess (e.g., differential biology), many will still assume that the diagnosis should indeed split apart individuals with very different underlying biology behind the phenotype. Evidence has been mounting in recent years suggesting that early language issues are quite important stratifiers, both from a clinical standpoint, but also in terms of underlying biology (3, 35–49). At what point should the field reflect back on whether such changes some several decades back were warranted?

Without being aware of these changes and the biases that they may represent in different subfields within autism research, the field can be quite a confusing array of findings that may be overgeneralized to the entire population. While some autistic individuals have problems in both verbal and non-verbal cognitive abilities, higher degrees of imbalance between these domains are common in autism (50–53). This is particularly important to consider given that the subset of individuals whom are minimally verbal and intellectually disabled individuals are much harder to test. Thus, a vast majority of neuroimaging, behavioral, and cognitive studies heavily rely on verbal individuals with intact intellectual functioning (35, 54). Findings from such studies do not necessarily represent what may be generalized to all autistic individuals, but rather to this subset of verbal and average-to-high intelligence individuals. Many individual studies suggest this as a caveat for interpreting their results. However, such caveats can be commonly overlooked when the literature is assessed in aggregate and may thus, give off the impression that the data represents effects that generalize to the entire population as a whole. When heterogeneity in early language and intellectual ability is examined as a factor of interest, remarkable differences can be detected (37–40, 45, 46). This indicates that neglect of studying minimally verbal and intellectually disabled individuals may mask very important differences within the autism population. Conversely, blood and other biological samples that allow for DNA extraction can be collected on these “harder to test” individuals, and as such, this literature may be biased in the other direction from neuroimaging, cognitive, and behavioral research on autism. Although autism is highly heritable and much of that inherited risk may reside in a polygenic mixture of small-risk commonly occurring variants, most of the more prominent findings in the autism genetics literature are restricted to rare *de novo* variants that affect a small minority of all autistic cases, and which nearly all co-occur with intellectual or other types of developmental disabilities at the phenotypic level (47–49, 55). Thus, it appears that different research literature in autism research may be inadvertently revealing aspects that are more pertinent to subsets of the population tied to this key change point in the shift from DSM-III to DSM-III-R. Without key attention focused on this nuance, the research may come off as being over-generalized to all within the autism population.

Although some of these features regarding differences in intellectual ability and early language were attempted to be retained in DSM-IV (e.g., Asperger’s Syndrome vs. autism), they have now largely been kicked to the wayside in DSM-5 (i.e., specifiers), while other features have seemed to stick. Rather than a symptom triad (pre-DSM-5), we now have a core symptom dyad of social-communication (SC) and restricted repetitive behavior (RRB) difficulties. SC and RRB tend to be the common denominators that all autistic individuals can be characterized by. However, because they are the common denominators that go into the diagnosis itself, and perhaps because they have stuck where other features have not, SC and RRB are also endowed (either explicitly or implicitly) with extra face validity for being the most important or essential (core or hallmark) features of autism. But are these the most important and essential elements/features of interest? Whether the current conception delineates what is truly core, important/essential features is likely a debate that will continue.

## “All Models are Wrong – but Some are Useful”: Being Mindful of Purposes, Goals, and Optimization

In autism research we must be mindful that there are numerous end goals or purposes behind different types of studies. Certainly all are concerned with the diagnostic label of autism, but they may be interested in whether that diagnostic label is important for explaining a variety of very different types of things. We believe it is important to be mindful here that we are essentially building models for explaining various different phenomena of interest. These models may incorporate the diagnostic label of autism versus non-autism as one of the explanatory features (e.g., the case-control model), but may also incorporate other features. On this topic, it is important to remember the statistical aphorism that “all models are wrong, but some are useful” (56). A model’s utility or usefulness is its power over explaining why variability in the phenomenon of interest occurs. Being mindful of this, we must also understand that the diagnosis itself is already an optimization for a very specific set of phenomena of interest – that is, the label of autism versus non-autism is maximally sensitive and specific for explaining variability in exemplar types of SC and RRB behaviors (57). Because of this, we can then invert the model and say that if the end purpose or goal was to predict or explain variation in the labels of autism versus non-autism, we would need a set of exemplar features of SC and RRB that are maximally useful for explaining autism versus non-autism label variation. Having specified that the label of autism already has been optimized for a specific goal or end purpose, if we turn our attention to explaining other phenomena of interest – that is, biology, outcomes, or treatment (BOT) response – then we should be fully aware of the reality that the diagnostic label of autism may not be guaranteed to explain these other phenomena very well, no matter how much we hope or assume them to be so. The diagnostic label of autism has already been optimized for a certain end goal or purpose at the level of behaviors within SC and RRB domains, and there is no guarantee that the label will also be highly useful outside of this scope.

## The BOT Objectives – Biology, Outcomes, and Treatment

Besides explaining the behavioral phenotype of autism (e.g., SC and RRB domains), what else should we care about explaining? There are numerous directions one could go here. However, the field already has some top priorities in this realm, regarding the ability to explain variability in differential biology, outcomes and treatment responses. We would call this subset of translational research objectives the “BOT objectives.” Are the BOT objectives aligned with the behavioral diagnosis of autism? If we were to assume a simple one-to-one linear mapping of biology to behavior, and vice versa, perhaps we could expect that biology exists that is indeed linked to the core hallmark SC and RRB features of autism. So far, we have not yet discovered such a mapping between biology and behavior in autism. Perhaps we haven’t been looking in the right places, or perhaps we aren’t yet equipped with the right tools to discover such a mapping, but perhaps we should also be prepared to accept that such an assumption is untenable as well. Thus, although the diagnosis is automatically endowed with some validity related to these BOT objectives, the reality is that the diagnosis was never designed to be optimal for explaining them. Rather, the diagnosis is optimized to explain phenomena in SC and RRB at the behavioral level. Thus, we should resist the assumption that the diagnosis should also be relevant for the BOT objectives until proven otherwise. If over time the science shows that the diagnostic label of autism may not be optimal for explaining the BOT objectives, we should then start seeking other types of models with other kinds of features that better explain the variability in differential biology, outcomes and treatment. We believe the field is already ready for this type of change (58) and we would issue a call-to-action to support the exploration of multiple other types of definitions that could be more useful for explaining BOT objectives within the autism population. However, such a call-to-action does not mean the diagnosis of autism has failed. The diagnostic label will always have the validity in being optimal for maximizing clinical consensus based on behavior. But we should be careful not to give it too much external validity with regards to other objectives that it was never optimally defined to explain in the first place.

## The Tree Analogy

We would like to conclude our discussion on ways to rethink autism with an analogy about the concept of “trees.” We turn to this analogy not because it represents a foolproof analogy that is 100% similar in every way. There are likely many extraneous aspects of this analogy that may not be best. However, we highlight here some specific similarities in this analogy to make salient a couple of key points that may shake our deeply held assumptions about concepts like autism.

In this analogy, we will ask the simple question of “what are trees?” This is meant to be analogous to the question of “what is autism?” On the surface, we might think this is a relatively easy question to answer, because we should all have a strong common sense understanding of what trees are. Indeed this common sense understanding of the concept of “tree” is so strong that if we go back to historical roots, we can find language that describes trees in Sanskrit and ancient Greek. This indicates that our ancestors must have valued and distinguished these types of plants so much that they thought it was pertinent to give a unique word to them. Because there is such a strong commonsense understanding of “tree,” could we distill an actual consensus definition as to what are the defining characteristics of trees that set them apart from other things in the plant kingdom? Below is a layperson’s consensus criteria on what is the most agreed upon definition (from Kim Coder’s outreach article entitled “What is a Tree?”) (59).

– Made mostly of woody substance.
– Has an erect, self-supporting, single unbranched trunk, or stem.
– Growth is perennial (throughout the year).
– Large and tall when fully mature.
– Has an elevated crown or branches.

The criteria above represent a consensus amongst a variety of dictionary, encyclopedia, botanical glossary, and ordinance/regulatory definitions of trees. This type of “consensus” definition has parallels to clinical consensus definitions based on behavior that we have about autism. The original concept behind the diagnosis was one based on observational and clinical consensus. Indeed, many experienced clinicians may have a similar strong common sense understanding of what the autism phenotype looks like, and as such, may not need much time while assessing some children to identify this autism prototype (4). While there is a difference in who makes the consensus definition (e.g., all people in the case of trees, versus specialists in the case of autism), the similarity we wish to underscore here is that both trees and autism have a consensus definition based on observable features (physical features in the example of trees, behavioral features in the example of autism).

Now that we have our consensus definition, let’s take the analogy one step further and assume that our definition of “tree” has face validity and value outside of the original context where the definition was optimized (e.g., on the basis of observable physical characteristics). Could we assume that the consensus definition of “tree” has validity with regard to how botanists would taxonomically characterize plants? In other words, because we have this strong consensus definition that trees are indeed a distinct “thing” in nature, would botanical taxonomies respect that and also distinguish trees as a specific scientific grouping separated from other plants? The answer here is simply “no.” Taxonomically, “trees” do not have a distinct scientific grouping. Rather, some trees are grouped into a cluster for flowering plants where seeds are encapsulated, called angiosperms (e.g., fruit trees), while the other types of trees are grouped into a class called gymnosperms, which have their seeds exposed (e.g., pine trees). Therefore, just because trees grow larger and taller than their other plant relatives in the same category, does not really matter, at least for an objective scientific definition. A blueberry bush and an apple tree come from the same angiosperm family and are not necessarily distinguished by the fact that apple trees fall within our definition of tree above, but blueberry bushes do not. Indeed, the point we are emphasizing here is that the concept of “trees” is a consensus definition historically defined in language by our human ancestors and which has carried on today. There are very good reasons to hold onto this definition, as it has value for labeling a specific type of plant that we all value culturally and wish to distinguish from other types of plants. However, although there is that consensus definition of “tree,” it does not correspond well with other taxonomic classifications of plants. Other taxonomic classifications of plants are optimized in other ways that do not correspond well to optimization within the consensus model for “tree.” Thus, the concept of tree is very salient to most of us and it may be very hard to shake the idea that scientifically, trees are not “one thing” that exists in nature. This point is emphasized to underscore the fact that although we might all be able to agree on a set of defining criteria, that by no means gives us license to assume that such a “thing” actually exists out in nature as an objectively defined “thing” and that all other connotations about underlying biology, etc., should follow from the initial consensus definition.

We can take a final step further in this analogy by considering the defining core or non-core characteristics of “trees” and drawing parallels to the diagnosis of autism. For example, let us say that the feature of growing large and tall when fully mature is akin to the RRB domain in autism, while the trunk or stem of a tree might be akin to the SC domain in autism. Here are two central defining features of trees and autism that are very well evident. Now let us take the seeds trees produce, along with the encapsulating tissue around it (e.g., fruit) and let us say that this is analogous to early language issues. Not all individuals with autism have early language issues, but some do. Not all trees bear fruit, but some do. A characteristic such as bearing fruit is not a core characteristic of a tree, because not all trees bear fruit, and also because many non-tree plants can bear fruit as well. Similarly, early language issues are no longer a core feature of autism (as they were in DSM-III) because the rationale is that not all individuals with the other core features have early language issues, and most crucially, because many non-autistic individuals can have substantial early language issues. By drawing this analogy, we would like to point out a distinction of importance. Fruit bearing trees are indeed a different class of plant altogether (i.e., an angiosperm) and such plants are so heavily differentiated from other trees falling into the gymnosperm category as to not be considered together, even despite the fact that, for example, a pine tree and an apple tree meet all the other core features of being a “tree.” By focusing on the core elements that are characteristic of autism, and marginalizing the importance of other non-core features, are we missing important aspects that would help us to derive a different definition or set of labels that is more tailored to elucidating differences that are considered to be of high-importance (e.g., BOT objectives)? This example is one of many that could be drawn in this type of “tree” analogy, and we offer it up as a potential thought experiment to help readers challenge their assumptions about autism and what is held to be of most importance, given a specific end goal/purpose/objective.

## Conclusion

In conclusion, we think the time is ripe to actively have the field reconsider or rethink their assumptions and strongly held core beliefs about autism. In doing such a “rethink,” we should consider that the single diagnostic definition we currently possess need not be the only or most important way of defining the autisms. Indeed, we may need multiple different types of definitions or classification structures (i.e., models) that are tailored to different end goals/objectives. We hope that we have been able to make more salient that the diagnostic model currently in place is there to be optimal for a specific type of phenomena and end goal and that for other purposes or objectives, other models may be needed. As we consider other models, we may need to let go of what we believe are the core versus non-core features of the current diagnostic model, and think about other ways to optimally explain autism in terms of a variety of alternative, yet important objectives (e.g., BOT objectives).

## Author Contributions

Both authors listed have made a substantial, direct, and intellectual contribution to the work, and approved it for publication.

## Conflict of Interest

The authors declare that the research was conducted in the absence of any commercial or financial relationships that could be construed as a potential conflict of interest.

## Publisher’s Note

All claims expressed in this article are solely those of the authors and do not necessarily represent those of their affiliated organizations, or those of the publisher, the editors and the reviewers. Any product that may be evaluated in this article, or claim that may be made by its manufacturer, is not guaranteed or endorsed by the publisher.
